# Perborate Activated Peroxymonosulfate Process for Improving the Coagulation Efficiency of *Microcystis aeruginosa* by Polymeric Aluminum Chloride

**DOI:** 10.3390/molecules29225352

**Published:** 2024-11-14

**Authors:** Fan Chen, Lu Li, Shunfan Qiu, Shiyang Chen, Lingfang Yang, Lin Deng, Zhou Shi

**Affiliations:** 1 Hunan Engineering Research Center of Water Security Technology and Application, College of Civil Engineering, Hunan University, Changsha 410082, China; fanchen123@hnu.edu.cn (F.C.);; 2 Key Laboratory of Building Safety and Energy Efficiency, Ministry of Education, Hunan University, Changsha 410082, China; 3 China Machinery International Engineering Design & Research Institute Co., Ltd., Changsha 410021, China

**Keywords:** cyanobacteria, perborate, peroxymonosulfate, polymeric aluminum chloride, disinfection by-products

## Abstract

In this study, the sodium perborate (SP)-activated peroxymonosulfate (PMS) process was used to enhance the coagulation efficiency of cyanobacteria with polymeric aluminum chloride (PAC), aiming to efficiently mitigate the impact of algal blooms on the safety of drinking water production. The optimal concentrations of SP, PMS, and PAC were determined by evaluating the removal rate of OD_680_ and zeta potential of the algae. Experimental results demonstrated that the proposed ternary PMS/SP/PAC process achieved a remarkable OD_680_ removal efficiency of 95.2%, significantly surpassing those obtained from individual treatments with PMS (19.5%), SP (5.2%), and PAC (42.1%), as well as combined treatments with PMS/PAC (55.7%) and PMS/SP (28%). The synergistic effect of PMS/SP/PAC led to the enhanced aggregation of cyanobacteria cells due to a substantial reduction in their zeta potential. Flow cytometry was performed to investigate cell integrity before and after treatment with PMS/SP/PAC. Disinfection by-products (DBPs) (sodium hypochlorite disinfection) of the algae-laden water subsequent to PMS/SP/PAC treatment declined by 57.1%. Moreover, microcystin-LR was completely degraded by PMS/SP/PAC. Electron paramagnetic resonance (EPR) analysis evidenced the continuous production of ${SO}_{4}^{•-}$, •OH, ^1^O_2_, and $O_{2}^{•-}$, contributing to both cell destruction and organic matter degradation. This study highlighted the significant potential offered by the PMS/SP/PAC process for treating algae-laden water.

## 1. Introduction

The proliferation of global warming and excessive nutrients, particularly nitrogen and phosphorous, results in the frequent occurrence of cyanobacteria blooms in lakes, reservoirs, and rivers. This has emerged as a significant environmental concern worldwide [1,2]. Cyanobacteria blooms not only directly impact the survival of aquatic organisms but also release hazardous substances such as microcystins, taste and odorous compounds, and endotoxins. These pose a serious threat to human health and drinking water safety [3,4]. Consequently, there is an urgent need to explore viable and effective techniques for the elimination and inactivation of cyanobacteria.

The coagulation process, which aims to remove suspended solids such as colloids and dissolved organic matter (DOM), serves as the initial treatment step in conventional surface water treatment processes. However, due to the negative charge on algal cells’ surfaces and the presence of electrostatic repulsion, hydrophilic properties, and steric effects, their aggregation is hindered, thereby reducing the coagulation efficiency [5]. However, the reaction between inorganic coagulants and algal organic matter (AOM) can result in the formation of chelate complexes, which diminishes the coagulation effectiveness of the coagulants and consequently increases the demand for coagulant dosage to achieve comparable efficiency [6,7]. In recent years, various pre-oxidation strategies, including utilizing permanganate, ozone, chlorine, ferrate, and hydrogen peroxide, have been documented for their potential in enhancing the coagulation of algal cells [7]. Oxidants can induce the desorption of organic coatings from the surface of cells, thereby reducing the cell stability and improving the coagulation efficiency [8]. However, the combination of ozone and chlorine can induce direct lysis of algal cells, leading to the release of intracellular organic matter (IOM) through excessive oxidation. This process ultimately enhances the formation potential of disinfection by-products (DBPs) [9]. Furthermore, the presence of permanganate and ferrate residues, which possess chemical toxicity, can have a detrimental impact on water quality [10].

Advanced oxidation processes based on sulfate radical (${SO}_{4}^{•-}$) and hydroxyl radical (•OH) have attracted extensive attention as a pre-oxidation process prior to coagulation for the enhancement of algae removal [7,11,12]. The synergistic effects of deactivating viable algae cells, altering the surface characteristics of algae cells, and decomposing and adsorbing dissolved organic carbon (DOC) all contribute to improved coagulation efficiency [11,12,13]. In contrast with •OH, ${SO}_{4}^{•-}$ is more preferred due to its longer half-life (30–40 μs), higher redox potential (2.5–3.1 V), and high selectivity toward organic compounds within a wide pH range [14,15]. Peroxymonosulfate (PMS, ${HSO}_{5}^{-}$) is regarded as a potential oxidant for ${SO}_{4}^{•-}$ generation as its possible final products are ${SO}_{4}^{2-}$, H_2_O and O_2_, which makes it desirable for water treatment [2,5,6,12,16]. For instance, an Fe(II)/PMS-enhanced coagulation process achieved 94.3% removal of cyanobacteria with 20 mg/L PMS and 4.5 mg/L Fe(II) at an initial concentration of 1.0 × 10^6^ cells/mL [7]. The removal efficiency of algae using a PMS-enhanced Fe(III) coagulation technique reached 97.5% in the presence of 1 mM PMS and 0.18 mM Fe(III), which was 1.5 times higher than that achieved using Fe(III) coagulation alone [17]. Approximately 98.2% of algae cells were eliminated via treatment with UV/persulfate (PS) for 2 h at a PS dosage of 1500 mg/L (UV density: 1.25 mW/cm^2^) [18]. Although significant progress has been made in applying ${SO}_{4}^{•-}$-based pre-oxidation technologies to enhance coagulation efficiency in algae-laden water treatment, the practical implementation is greatly hindered by water acidification caused by PMS by-products and the hydrolytic reaction of Fe(II) or Fe(III) [2,10,11,16,17,19]. Therefore, it is crucial to utilize environmentally friendly, highly efficient, and cost-effective chemicals as activators for PMS-based pre-oxidation processes aimed at algae removal. Sodium perborate (SP) is an air-stable oxidant that readily dissolves in acid pH conditions to produce H_2_O_2_. Our previous study demonstrated that PMS activation using peroxyborate not only achieved superfast degradation for bisphenol A (BPA) but also significantly mitigated the decline in solution pH [20]. Nevertheless, the current knowledge regarding the utilization of PMS/peroxyborate for treating water contaminated with algae remains limited. For instance, it is still unknown whether this process effectively enhances the coagulation efficiency of algae cells. Furthermore, the mechanisms underlying their inactivation are yet to be determined. Additionally, it is unclear whether this method can simultaneously remove algae cells and control DBP formation. Moreover, further investigation is required to determine which reactive oxygen species contribute to the removal of algae cells. All these inquiries warrant further exploration.

Herein, we employed the peroxyborate-activated PMS process to enhance the coagulation efficiency of cyanobacteria algae using polymeric aluminum chloride (PAC), a commonly used flocculant in drinking water treatment plants. The specific objectives of this study were (1) to investigate the impact of PMS dose, peroxyborate dose, and PAC dose on the removal efficiency of cyanobacteria; (2) examine the integrity of algae cells; (3) unveil the possible mechanisms involved in the enhancement of algae removal; and (4) compare variations in DBP formation during chlorination.

## 2. Results and Discussion

### 2.1. Comparison of Different Treatment Processes on Cyanobacteria Removal

**Figure 1:**
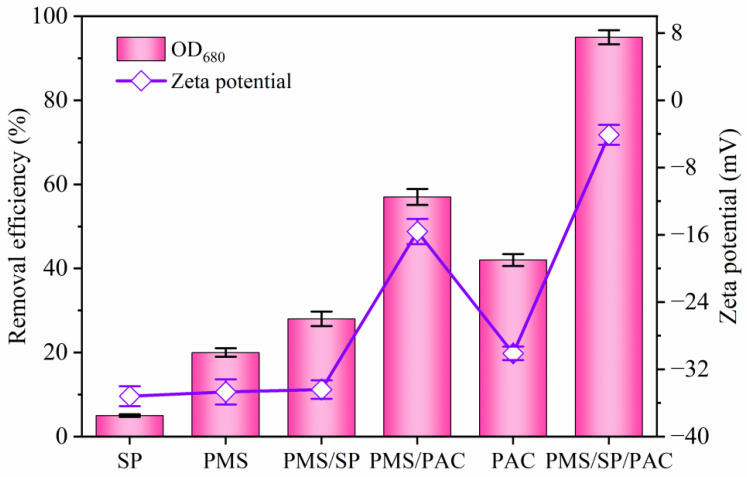
Comparison of different processes on cyanobacteria removal (initial algal cell density: 5.0 × 10^6^ cells/mL, SP dosage: 1 mM, PMS dosage: 3 mM, PAC dosage: 10 mg/L).

The cyanobacteria removal rates using PAC, PMS, SP, PMS/SP, PMS/PAC, and PMS/SP/PAC were comparatively investigated. As shown in Figure 1, both SP (1 mM) and PMS (3 mM) exhibited limited efficacy in eliminating cyanobacteria cells with OD_680_ removal rates of 5.2% and 19.5%, respectively. In comparison, the addition of 10 mg/L PAC realized a removal efficiency of 42.1%, primarily related to the exceptional coagulation performance. The presence of PMS also marginally improved the removal rate to 55.7% in the binary PMS/PAC system due to the generation of reactive oxygen species that could destroy cyanobacteria cells in solution. Meanwhile, simultaneous injection of PMS and SP only resulted in a modest algae removal rate of 28%. These findings underscored the significant role played by coagulants in removing algae cells. Notably, when SP was introduced into the PMS/PAC system under identical experimental conditions, it yielded the highest OD_680_ removal rate (95.2%).

The ternary PMS/SP/PAC system significantly enhanced the aggregation of cyanobacteria cells, as evidenced by the observed variation in the zeta potential during the removal process. In the control group, without the addition of any chemicals, the algae cell surface exhibited a highly negative charge with a zeta potential of −40.5 mV, suggesting that the raw algae-laden solution was stable due to strong electrostatic repulsion existing between algae cells. As shown in Figure 1, treatment with SP, PMS, and PAC resulted in gradual increases in zeta potential values to −35.2, −34.7, and −30.1 mV, respectively. However, when using the PMS/SP/PAC system, there was significant improvement as it sharply increased to −4.1 mV. It is worth noting that this change in zeta potential was positively correlated with algae removal efficiency.

The decrease in the absolute value of the zeta potential played a critical role in enhancing the removal of algae cells [21]. In this study, PMS/SP/PAC treatment effectively reduced the absolute value of the zeta potential, which led to a decline in surface charge on algae cells, resulting in weakened electrostatic repulsion and improving coagulation efficiency.

### 2.2. Effects of PMS, SP, and PAC Dosage on Cyanobacteria Removal

**Figure 2:**
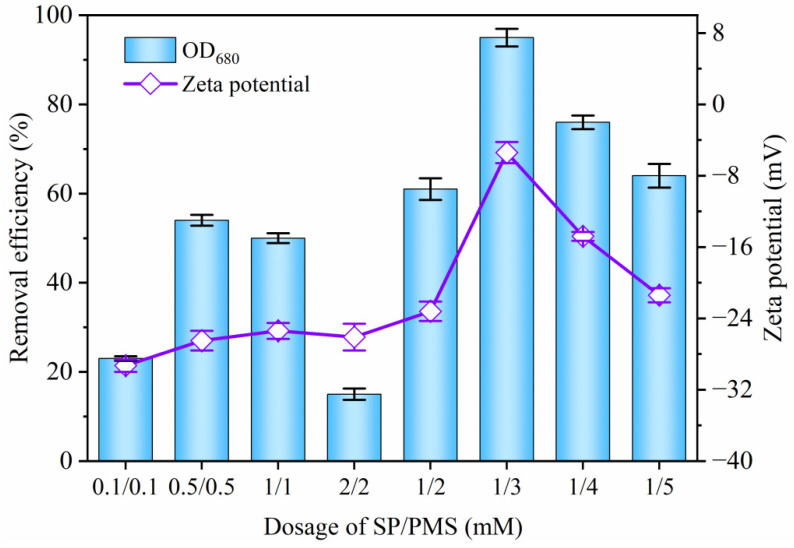
Effect of SP and PMS dosage on cyanobacteria removal using PMS/SP/PAC treatment (initial algal cell density: 5.0 × 10^6^ cells/mL, PAC dosage: 10 mg/L).

The effects of PMS and SP dosage on the removal of cyanobacteria using PMS/SP/PAC were further investigated at a controlled PAC dosage of 10 mg/L. As shown in Figure 2, when the concentration of SP and PMS in the cyanobacteria-laden solution was 0.1 mM, the removal rate of OD_680_ was only 23.2% with a zeta potential of −29.3 mV. However, increasing the dosage to 0.5 mM resulted in an enhanced removal efficiency of 54.5%, suggesting that the concentration levels played a crucial role in eliminating cyanobacteria cells. Nevertheless, further elevating the SP and PMS dosage to 2 mM sharply decreases the OD_680_ removal rate to only 15.1%. Considering these findings, a concentration level of 1 mM for SP was selected for subsequent tests.

**Figure 3:**
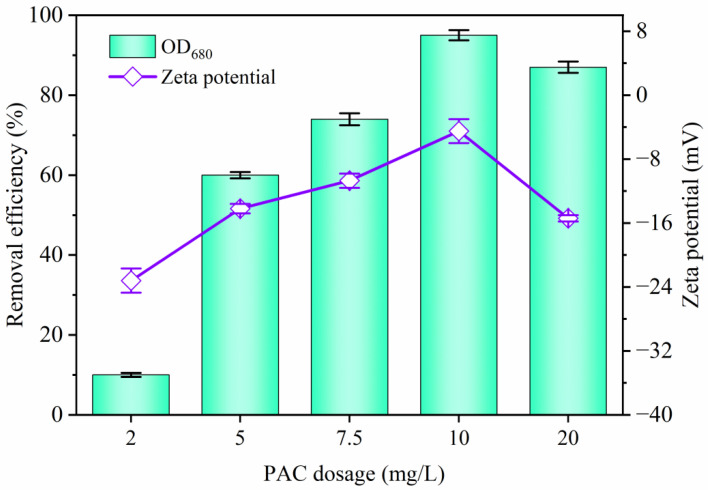
Effect of PAC dosage on cyanobacteria removal using PMS/SP/PAC treatment (initial algal cell density: 5.0 × 10^6^ cells/mL, SP dosage: 1 mM, PMS dosage: 3 mM).

Figure 2 also displays the contribution of PMS concentration from 1 to 5 mM to the removal of cyanobacteria. It is evident that the removal rate of OD_680_ significantly increased from 49.5% to 95.2% with an increase in PMS dosage from 1 to 3 mM. The elevated PMS concentration improved the production of ${SO}_{4}^{•-}$ and •OH, leading to cell destruction and alteration of the surface properties. However, it gradually dropped to 64.5% with a further increment of PMS dosage from 3 to 5 mM due to excessive PMS causing difficulty in cell precipitation, resulting in a decline in removal efficiency. Consequently, 1 mM of SP and 3 mM of PMS were selected for subsequent experiments. Furthermore, the influence of PAC dosage on cyanobacteria removal was also studied. As shown in Figure 3, the algae removal rate using PMS/SP/PAC was highly dependent on the PAC dosage employed. When only adding 2 mg/L of PAC, the removal efficiency of OD_680_ was less than 10%, indicating insufficient coagulation performance at this low PAC concentration level. However, when increasing the PAC concentration to the levels of 5 mg/L, 7.5 mg/L, and 10 mg/L, the removal efficiency improved to 60.1%, 74.5%, and 95.2%, respectively. Yet, it slightly declined to 87.2% when an excessive amount of PAC (20 mg/L) was presented.

### 2.3. Cellular Integrity Analysis

The release of K^+^ is commonly employed as an indirect indicator of cell disruption, given that K^+^ plays a crucial role in the biosynthesis of cytoplasmic membranes. As indicated in Table 1, the control group, without the addition of any chemicals, exhibited a K^+^ release ratio of 1.4%, signifying intact cell membrane integrity. In contrast, when the algae-laden water was treated with PMS/SP/PAC, there was a significant increase to 96.4%. The large amount of K^+^ release clearly indicated the severe damage to algae cell membrane integrity [13].

**Figure 4:**
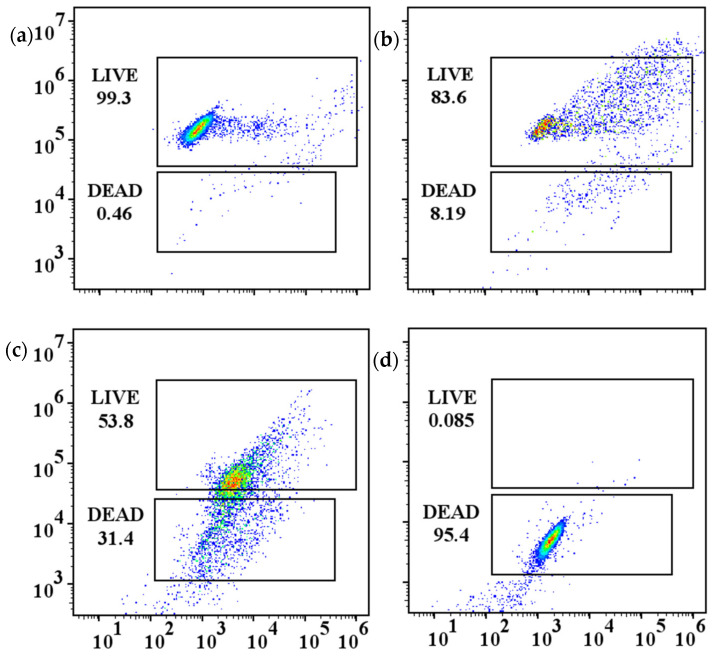
Cell integrity of algae treated with (**a**) control, (**b**) PAC, (**c**) PMS/PAC, and (**d**) PMS/SP/PAC (initial algal cell density: 5.0 × 10^6^ cells/mL, SP dosage: 1 mM, PMS dosage: 3 mM, PAC dosage: 10 mg/L).

Furthermore, a flow cytometer was used to visualize the cell integrity of algae treated with PAC, PMS/PAC, and PMS/SP/PAC, as depicted in Figure 4. The dead and live regions represent damaged and undisrupted cell populations, respectively. In the control group, approximately 99.3% of cyanobacteria cells were found to be intact (Figure 4a), indicating that the majority of cells remained undamaged without any treatment. When the algae-laden water was exposed to PAC, there was a slight decrease in undisrupted cells within the live region to 83.6% (Figure 4b), suggesting that individual PAC coagulation alone did not completely eradicate algae cells. As shown in Figure 4c, the presence of PMS resulted in the proportion of live cells being 53.8%, which can be attributed to reactive substances generated by the presence of PMS. Notably, PMS/SP/PAC exhibited superior efficacy in damaging algae cells with a dead region percentage of 95.4% (Figure 4d). Simultaneously, a minimal signal was observed within the live region, implying that this ternary system effectively further oxidized nucleic acid due to the generation of reactive species.

### 2.4. DBP Formation Potential in Chlorination Following PMS/SP/PAC Treatment

The released DOM from cyanobacteria cells is reported to make a significant contribution to the precursors of known DBPs [8]. To better assess the impact of PMS/SP/PAC on algae-laden water treatment, potential carbonaceous DBPs, including trichloromethane, 1,3-dichloropropane, and 1,2-dichloropropane as well as nitrogenous DBPs such as dichloroacetonitrile and trichloronitromethane produced during the post-chlorination were analyzed. The DBP formation experiments were performed in sealed 40 mL amber bottles containing sodium hypochlorite in dark conditions at a temperature of 25 °C. These experiments followed the degradation process with a one-day incubation period. Ascorbic acid was used to terminate the production of DBPs in water samples. Thereafter, the potentially formed DBPs were detected using the method described in the aforementioned section.

**Figure 5:**
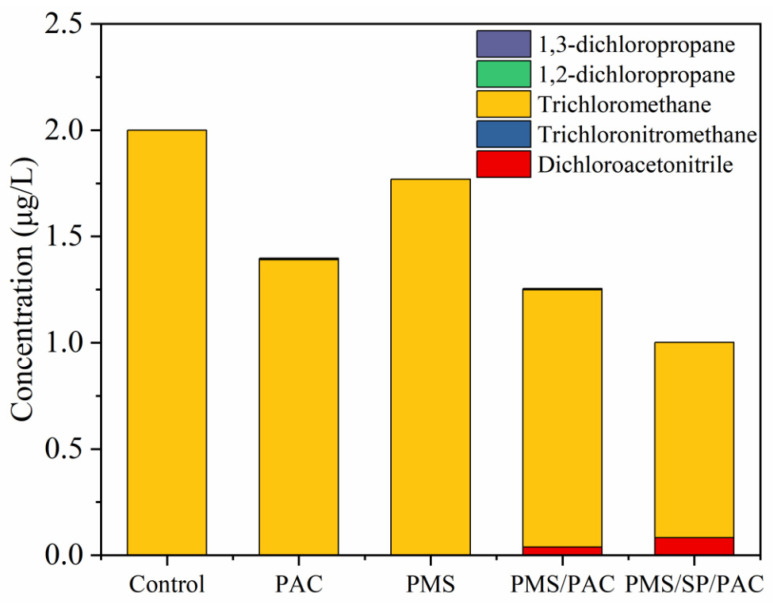
DBP yield from different treatments of cyanobacteria under chlorination in DI water (initial algal cell density: 5.0 × 10^6^ cells/mL, SP dosage: 1 mM, PMS dosage: 3 mM, PAC dosage: 10 mg/L).

As shown in Figure 5, the presence of 1,3-dichloropropane, 1,2-dichloropropane, and trichloronitromethane was not detected in any of the water samples. Initially, the concentration of trichloromethane in the untreated algae-laden water was measured at 2.1 μg/L. However, this concentration decreased after treatment with PAC, PMS, PMS/PAC, and PMS/SP/PAC. Notably, it sharply decreased to 0.9 μg/L when treated with PMS/SP/PAC. Nitrogenous DBPs of dichloroacetonitrile were only observed in PMS/PAC and PMS/SP/PAC systems at concentrations of 0.03 and 0.08 μg/L, respectively. Overall, compared to the control group, there were significant reductions in DBP concentrations during post-chlorination in the PMS/SP/PAC system by approximately 57.1%, indicating the effectiveness of this proposed system in mitigating DBP formation.

### 2.5. Microcystin-LR Release After PMS/SP/PAC Treatment

**Figure 6:**
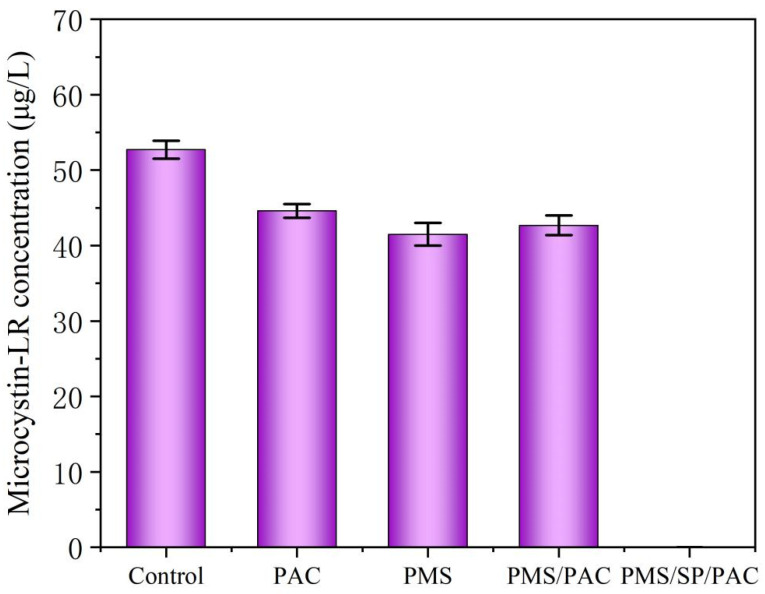
Microcystin-LR elimination using different treatment processes in the removal of cyanobacteria (initial algal cell density: 5.0 × 10^6^ cells/mL, SP dosage: 1 mM, PMS dosage: 3 mM, PAC dosage: 10 mg/L).

The release of microcystin-LR during the reaction is a significant concern regarding the deactivation of cyanobacteria for water safety. Therefore, we recorded the leakage of microcystin-LR after PMS/SP/PAC treatment to investigate whether reactive species generated by this process can effectively degrade microcystin-LR released by cyanobacteria to mitigate harm to the environment [22]. As shown in Figure 6, the initial concentration of dissolved microcystin-LR in the untreated algae-laden water was 52.6 μg/L. The addition of PAC led to a decrease in microcystin-LR concentration to 44.2 μg/L. Similar to the result in Figure 1, 3 mM of PMS also could not effectively remove microcystin-LR. This observation agreed well with the previous work [6,11]. By contrast, after treatment with PMS/SP/PAC, no detectable levels of microcystin-LR were observed, suggesting the significant potential of applying the proposed ternary system to remove microcystin-LR. The permissible concentration of microcystins-LR in China is 1 μg/L regulated by ‘Standards for drinking water quality (GB 5749-2022)’ [23,24]. The Environmental Protection Agency (US EPA) has established a health warning value for microcystin in drinking water with a maximum concentration of 0.3 μg/L for infants and 1.6 μg/L for school-age children and adults [25]. The World Health Organization (WHO) recommends that the concentration of microcystin in drinking water must be below 1 μg/L [26].

Based on the aforementioned analysis, we are reasonably confident that the proposed PMS/SP/PAC process surpasses PAC, PMS, and PMS/PAC in terms of its ability to effectively eliminate algae cells in water. Additionally, it also reduces the potential formation of DBPs and efficiently decomposes microcystin-LR released from cyanobacteria cells.

### 2.6. Mechanisms Investigation

**Figure 7:**
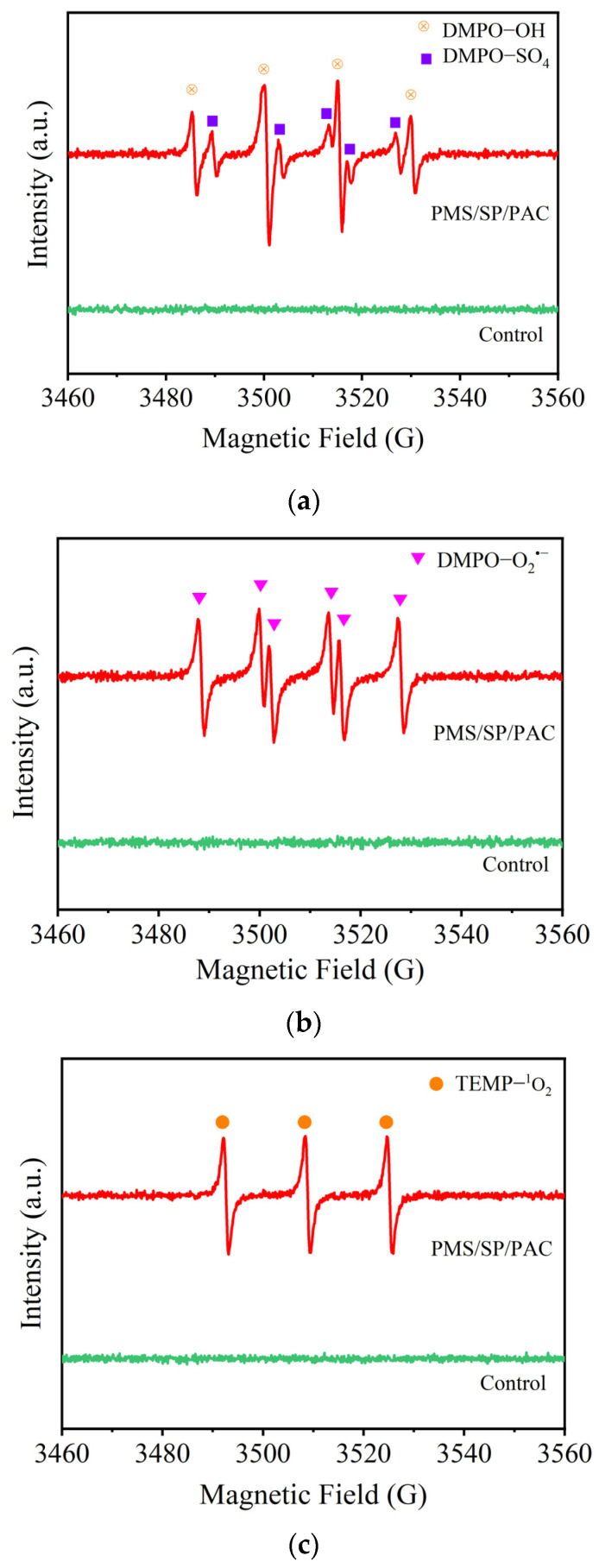
The EPR spectra of (**a**) DMPO−OH and DMPO−SO_4_ adducts, (**b**) DMPO−$O_{2}^{•-}$ adducts, and (**c**) TEMP−^1^O_2_ adducts (initial algal cell density: 5.0 × 10^6^ cells/mL, SP dosage: 1 mM, PMS dosage: 3 mM, PAC dosage: 10 mg/L, [DMPO] = [TEMP] = 100 mM).

In this study, electron paramagnetic resonance (EPR) tests were performed to identify the possible formed reactive species in the removal of algae cells by the ternary PMS/SP/PAC system. EPR experiments were performed on a Bruker (Billerica, MA, USA) EMXplus EPR spectrometer: temperature 298 K; microwave frequency 9.823 GHz; microwave power 6.325 mW, and modulation amplitude 1.0 G. DMPO and TEMP were used as capture agents for radicals (${SO}_{4}^{•-}$, •OH, and $O_{2}^{•-}$) and non-radicals (^1^O_2_), respectively [1,17]. In the control group, only cyanobacteria mixed with the capture agents. No signals were detected in the control group. By contrast, as shown in Figure 7a, characteristic signals belonging to DMPO-OH and DMPO-SO_4_ adducts were detected, indicating the formation of ${SO}_{4}^{•-}$ and •OH [7,14]. A triplet signal (1:1:1) representing TEMP-^1^O_2_ adducts was observed in Figure 7b, evidencing the production of ^1^O_2_ [12,14]. Characteristic peaks of DMPO-$O_{2}^{•-}$ adducts indicated the presence of $O_{2}^{•-}$ (Figure 7c) [27]. Meanwhile, the peak intensity improved gradually along with an increase in the reaction time. This outcome suggested that ${SO}_{4}^{•-}$, •OH, ^1^O_2_, and $O_{2}^{•-}$ were continuously generated in the PMS/SP/PAC system, which could act directly on the cells via strong oxidation, thereby facilitating the removal of cyanobacteria [28].

## 3. Materials and Methods

### 3.1. Materials and Reagents

The cyanobacteria used in this study was *Microcystis aeruginosa* (No. FACHB-905), and it was purchased from the Institute of Hydrobiology, Chinese Academy of Science, and cultivated in a light growth incubator. The culture was maintained at a constant temperature of 25 ± 1 °C in a sterile environment, utilizing BG11 medium and following a 12 h light/12 h dark cycle. Weekly subculturing was performed to ensure the continuous exponential growth phase.

Peroxymonosulfate, sodium perborate (NaBO_3_·4H_2_O, SP), NaOH, and H_2_SO_4_ were purchased from Shanghai Aladdin Biochemical Ltd. (Shanghai, China). PAC and methyl alcohol (MeOH) were obtained from Sinopharm Chemical Reagent Co., Ltd. (Shanghai, China). Microcystin-LR (>95%) was purchased from Taiwan Algal Science Inc. (Taiwan, China). Trichloromethane, 1,3-dichloropropane, 1,2-dichloropropane, dichloroacetonitrile, trichloronitromethane, and 2,2,6,6-tetramethylpiperidine (TEMP) were obtained from Sigma-Aldrich Chemical Co., Ltd. (Saint Louis, MO, USA). 5,5-dimethyl-1-pyrrolidine-N-oxide (DMPO) was purchased from Dojindo Molecular Technologies (Rockville, MD, USA). SYTOX green nucleic acid stain was purchased from Invitrogen (Bend, OR, USA). All reagents were analytical or chromatograph grade and received for use without further purification. All solutions were prepared using ultrapure water (18.25 cm/Ω) produced using a Millipore Milli-Q gradient water purification system (Billerica, MA, USA). The stock solutions for PMS, SP and PAC were all freshly prepared prior to the experiments.

### 3.2. Experimental Procedure

The density of the diluted cyanobacteria solution was determined using a UV–vis spectrophotometer (Hitachi, U-3900, Tokyo, Japan) at a wavelength of 680 nm (OD_680_) since the OD_680_ value positively correlates with the concentration of cyanobacteria cells [1,18]. The quartz cell path length was 10 mm, and ultrapure water served as the control. Initially, the cyanobacteria solution was diluted with ultrapure water to obtain an algal intensity of 10^6^ cell/mL to simulate the actual density of cyanobacteria pollution in a natural algal bloom. The initial OD_680_ value was determined as 0.20 ± 0.02, equal to an algal intensity of ~5.0 × 10^6^ cells/mL.

Algae removal experiments were carried out using a programmable jar tester (MY3000-6H, MeiYu, China). Typically, 500 mL of cyanobacteria solution was transferred to a 1000 mL glass beaker. Subsequently, appropriate quantities of PMS, SP, and PAC were simultaneously added into the aforementioned solution while stirring. The coagulation process involved rapid stirring at 250 rpm for 1 min, followed by medium stirring at 100 rpm for 3 min and slow stirring at 40 rpm for 10 min. Afterward, the solution was allowed to settle statically for a duration of 20 min. Following sedimentation, a supernatant sample of 10 mL was withdrawn from the beaker, immediately filtered using a membrane filter with a pore size of 0.45 μm, and quenched using an excess amount of MeOH in order to measure the residual algae through OD_680_. The removal efficiency (*R*) was calculated using the equation of $R=\frac{\left(A_{0}-A_{t}\right)}{A_{0}}\times 100\%$, where *A*_0_ and *A*_t_ were the absorbance of cyanobacteria solution at 680 nm at 0 and t min, respectively. Note that all removal tests for cyanobacteria were carried out in triplicate.

The water samples were analyzed using a Malvern particle sizer (Malvern, S90, UK) with a potential cuvette (DTS1070) after undergoing various treatment processes. The change in zeta potential of the cyanobacteria was calculated using the formula $\Delta Z=Z_{0}$ − $Z_{t}$, where *Z*_0_ and *Z*_t_ were the zeta potential of samples before and after treatment, respectively.

### 3.3. Analytical Methods

To assess the damage caused by the PMS/SP/PAC process to cyanobacteria cells, the cell integrity before and after treatment was tested using a flow cytometer (Accuri C6, BD Biosciences, Milpitas, CA, USA) equipped with an argon laser emitter (Wavelength fixed at 488 nm) for fluorescence measurement [29]. SYTOX green nucleic acid staining was used to stain algae cells. As SYTOX can penetrate damaged cells and stain the nucleic acid to emit green fluorescence, green fluorescence in channel FL1 (530 nm) and red fluorescence in channel FL3 (630 nm) representing damaged and integrated cells, were collected with fluorescent filters and detectors, respectively [30]. Both of them can be separately collected to differentiate between the dead and live cells. Data were analyzed using Flowjo software v10.8.

The supernatant samples were centrifuged at 6000 rpm for 10 min and then filtered using a cellulose acetate membrane with a pore size of 0.45 μm for subsequent analysis. Since K^+^ is a key element in the cyanobacteria cell membrane, its release indirectly reflects the integrity of the cell membrane. The cell breakage on the basis of K^+^ release was determined using the formula $\frac{{C-C}_{P}-C_{0}}{C_{t}-C_{0}}\times 100\%$, where *C*_t_ was the total K^+^ concentration in the complete cell-broken samples with ultrasonic treatment for 30 min, *C*_P_ was the additional K^+^ concentration induced by the PMS presence in each experiment, and *C*_0_ and *C* were the concentrations of K^+^ before and after treatments by different processes, respectively. An inductively coupled plasma optical emission spectrometer (ICP-OES, OPTIMA 8000, PerkinElmer, Shelton, CT, USA) was used to measure the concentration of K^+^ in water samples.

The microcystin-LR was determined using a triple four-bar liquid chromatography–mass spectrometer (Shimazu LCMS 8050, Japan) according to ‘Determination of microcystins in water (GB/T 20466-2006) [31]’ established by General Administration of Quality Supervision, Inspection and Quarantine of the People’s Republic of China. The mobile phase of A was composed of 0.1% formic acid in ultrapure water, and the mobile phase of B was methanol. A C18 column (Anthena Uhplc C18 2.1 mm × 50 mm, 1.8 μm) was employed to separate the different compounds, and it was pre-activated using 20 mL of 100% methanol and 20 mL of ultrapure water. Firstly, 0.9 mL of the water sample was filtered through a glass fiber filter membrane (GF/C). Then, the filtrate passed through the C18 column and was successively washed with 50 mL of ultrapure water and 10 mL of 100% methanol. Thereafter, the eluent was evaporated in a water bath with a nitrogen stream and preserved in 1 mL of methanol at −20 °C for microcystin-LR concentration detection. The injection volume, flow rate, and column temperature were 10 μL, 0.4 mL/min, and 30 °C, respectively. MS data were obtained using a full scan from *m*/*z* 50 to 2000 in positive ion mode.

DBPs, including trichloromethane, 1,3-dichloropropane, 1,2-dichloropropane, dichloroacetonitrile, and trichloronitromethane potentially produced during the post-chlorination were extracted in a liquid–liquid procedure using methyl tert-butyl ether (MtBE) containing 1,2-dibromopropane as an internal standard and sodium sulfate. The DBP concentrations were determined using gas chromatography (GC, Agilent 7890A, Santa Clara, CA, USA) with an electron capture detector (ECD) and an Agilent DB-5 column (30 m × 0.25 mm × 0.25 µm). The injection port temperature was 170 °C. The temperature program started at 35 °C for 9 min, ramping to 40 °C at 2 °C/min and then ramping to 160 °C at 20 °C/min and holding at 160 °C for 10 min.

To identify the generation of reactive substances in the studied system, •OH and ${SO}_{4}^{•-}$ were captured using DMPO [20]. Specifically, 3 mM PMS, 1 mM SP, and 10 mg/L PAC were mixed with 5 mL of ultrapure water under ultrasound concussion. Then, 200 μL of the above mixture was withdrawn and mixed with 100 μL of DMPO (100 mM) rapidly. Thereafter, the obtained solution was introduced into a capillary tube and detected using an electron paramagnetic resonance (EPR) spectrometer (Bruker A300, Karlsruhe, Germany). DMPO was also used to capture $O_{2}^{•-}$. The analysis procedure was almost the same as the procedure described above; only 5 mL of ultrapure water was replaced by 5 mL of methyl alcohol. ^1^O_2_ was captured using TEMP (100 mM) through the same procedure for •OH and ${SO}_{4}^{•-}$. Two sets of parallel tests were performed to ensure the accuracy of the results.

## 4. Conclusions

The present study presents a ternary PMS/SP/PAC process for the efficient removal of cyanobacteria. The following conclusions can be drawn: (1) the proposed PMS/SP/PAC process achieved an OD_680_ removal rate of 95.2%, which was significantly higher compared to the individual treatments with PMS (19.5%), SP (5.2%), and PAC (42.1%); (2) the potential formation of DBPs during the subsequent chlorination was controlled to reduce the risk of harm to humans; (3) microcystin-LR in solution was completely eliminated by PMS/SP/PAC; (4) ${SO}_{4}^{•-}$, •OH, ^1^O_2_, and, $O_{2}^{•-}$ contributed to cell destruction and degradation of organic matter; (5) PMS/SP/PAC enhanced the aggregation of cyanobacteria cells due to the significant reduction in zeta potential. To be mentioned, the levels of ROS and other reactive species produced by the PMS/SP/PAC system may have the potential to affect aquatic biota (since they had the power to create algal cell destruction and oxidize organic matters); therefore, the ROS levels created by this system must be tested, under isolated and controlled conditions, on aquatic organisms before applying the method in the field.

## Figures and Tables

**Table 1:** The K^+^ release ratio in different processes after reaction.

	K^+^ Release Ratio
Control group	1.4%
10 mg/L PAC	14.1%
3 mM PMS	10.5%
1 mM SP	1.6%
3 mM PMS + 10 mg/L PAC	61.2%
3 mM PMS + 1 mM SP + 10 mg/L PAC	96.4%

## Data Availability

Data will be made available on request.
